# Thermal Conductance of the 2D MoS_2_/*h*-BN and graphene/*h*-BN Interfaces

**DOI:** 10.1038/srep43886

**Published:** 2017-03-06

**Authors:** Yi Liu, Zhun-Yong Ong, Jing Wu, Yunshan Zhao, Kenji Watanabe, Takashi Taniguchi, Dongzhi Chi, Gang Zhang, John T. L. Thong, Cheng-Wei Qiu, Kedar Hippalgaonkar

**Affiliations:** 1 Department of Electrical and Computer Engineering, National University of Singapore, Engineering Drive 3, 117583, Singapore; 2 Institute of High Performance Computing, #16-16, 1 Fusionopolis Way, Agency for Science, Technology and Research, 138632, Singapore; 3 Institute of Materials Research and Engineering, #08-03, 2 Fusionopolis Way, Agency for Science, Technology and Research, 138634, Singapore; 4 National Institute for Materials Science, Tsukuba, Ibaraki, 305-0044, Japan; 5 Optical Science and Engineering Center, Department of Electrical and Computer Engineering, National University of Singapore, 117583, Singapore

## Abstract

Two-dimensional (2D) materials and their corresponding van der Waals heterostructures have drawn tremendous interest due to their extraordinary electrical and optoelectronic properties. Insulating 2D hexagonal boron nitride (*h*-BN) with an atomically smooth surface has been widely used as a passivation layer to improve carrier transport for other 2D materials, especially for Transition Metal Dichalcogenides (TMDCs). However, heat flow at the interface between TMDCs and *h*-BN, which will play an important role in thermal management of various electronic and optoelectronic devices, is not yet understood. In this paper, for the first time, the interface thermal conductance (G) at the MoS_2_/*h*-BN interface is measured by Raman spectroscopy, and the room-temperature value is (17.0 ± 0.4) MW · m^−2^K^−1^. For comparison, G between graphene and *h*-BN is also measured, with a value of (52.2 ± 2.1) MW · m^−2^K^−1^. Non-equilibrium Green’s function (NEGF) calculations, from which the phonon transmission spectrum can be obtained, show that the lower G at the MoS_2_/*h*-BN interface is due to the weaker cross-plane transmission of phonon modes compared to graphene/*h*-BN. T*h*is study demonstrates that the MoS_2_/*h*-BN interface limits cross-plane heat dissipation, and thereby could impact the design and applications of 2D devices while considering critical thermal management.

Two-dimensional (2D) materials with atomic-scale thickness have drawn tremendous interest since the successful exfoliation of graphene^1^. Although graphene has a unique electronic bandstructure, which has led to interesting phenomena like massless Dirac fermion physics^2^,^3^,^4^ and anomalous quantum Hall effect^5^,^6^,^7^, its zero-bandgap makes it unsuitable for use as field effect transistors (FETs), despite some recent attempts to open a bandgap in graphene^8^,^9^,^10^,^11^,^12^. Transition metal dichalcogenides (TMDCs), including the semiconducting MX_2_ (M = Mo, W; X = S, Se, Te), on the other hand, have electronic bandgaps covering the visible region^13^. What is more interesting is that the bandgaps of TMDCs are thickness-dependent^14^. For instance, molybdenum disulfide (MoS_2_) has a bandgap ranging from 1.3 eV to 1.9 eV as the number of layers increases from single to bulk^15^. Hexagonal boron nitride (*h*-BN) is a good 2D insulator with a bandgap of 5.971 eV^16^. Because of its atomically smooth surface and dielectric nature, *h*-BN is widely used as a high quality substrate for graphene^17^,^18^ and TMDCs^19^,^20^,^21^. For MoS_2_ supported by *h*-BN, the carrier mobility increases by more than an order of magnitude compared to that of SiO_2_ supported MoS_2_, because *h*-BN effectively protects the MoS_2_ channel from Coulomb scattering by charged impurities in SiO_2_^21^, similar to what is observed for graphene FETs on *h*-BN^17^.

Compared to the numerous studies^13^,^14^,^22^,^23^,^24^,^25^,^26^,^27^ on the electronic and optoelectronic properties of 2D materials and the van der Waals heterostructures mentioned above, there is a paucity of research on their thermal properties in spite of their relevance to heat management, which is critical for maintaining optimal functionality of these devices^14^,^28^,^29^,^30^. In general, most thermal studies focus on the suspended configuration where the 2D material is isolated from a substrate to exclude the contribution from the substrate^31^,^32^,^33^,^34^,^35^. Although such measurements further our understanding of phonon interactions within the 2D system, they are of limited relevance to realistic device applications for which the supported configuration is more practical. The thermal properties of this supported configuration are still not well studied, and there are only few works reported on graphene^36^,^37^,^38^,^39^ and MoS_2_^40^,^41^ on various bulk substrates. TMDC flakes situated on another 2D material such as *h*-BN being the substrate, i.e., two 2D-material interface/heterostructure, is far less investigated, which is in great demand in the emerging heterostructures and optoelectronic devices involving stacked 2D materials. Compared to the suspended configuration where heat transport only occurs in the basal plane, the difficulty with the supported configuration comes from the three-dimensional (3D) nature of heat transport. Even though the in-plane thermal conductivities of the two materials involved could be obtained individually by suspending each sample across a measurement platform, the thermal conductivity changes when the samples are supported due to the suppression of the flexural mode phonons and interactions between the substrate and superstrate^36^,^37^,^42^. In order to deconvolve such a 3D problem, it is critical to know the interface thermal conductance.

Finite interface thermal conductance (G) was first observed by Kapitza at the copper and liquid helium boundary^43^, and was later explained by the acoustic mismatch model (AMM)^44^, which assumes that phonons reaching the interface undergo specular reflection or transmission following continuum mechanics for a perfectly smooth interface. However, subsequent experiments have shown that the AMM breaks down for phonons with frequency larger than 100 GHz^45^, therefore leading to the development of the diffuse mismatch model (DMM) for thermal phonons^46^. The DMM assumes that the transmission of phonons reaching the interface depends on the ratio of the phonon density of states of either side^46^. With the DMM, Reddy *et al*. calculated G for metal-semiconductor interfaces such as Al/Si, Al/Ge, Cu/Si and Cu/Ge using exact phonon dispersions^47^. Lyeo *et al*. measured G for an assembly of dissimilar materials using time-domain thermoreflectance (TDTR), exploring the lower bounds of G and found that such a lower bound actually lies within a narrow range of 8 to 30 MW/m^2^K^48^.

For supported 2D materials, although heat is dissipated predominantly in the vertical direction as compared to laterally^49^, the study of the vertical interface conductance, G, has just recently expanded to the regime of 2D materials. By Joule/optical heating graphene and measuring temperatures via Raman spectroscopy, Yue *et al*. measured G between epitaxial graphene and 4H-SiC to be 0.0189 MW/m^2^K, which is five orders of magnitude lower than that expected from their molecular dynamics (MD) simulations^50^. They attributed the much lower measured G to the significantly enhanced phonon scattering effect from the structural change at the interface caused by 1) the high stress induced by covalent bonds between graphene/SiC and 2) the thermal expansion mismatch caused by separation at the interface. With a similar technique, Chen *et al*. measured G between graphene and *h*-BN to be 7.4 MW/m^2^K^51^. Similarly, this result is still more than an order of magnitude smaller than the theoretically predicted value of 187 MW/m^2^K^52^, and is explained as a result of random lattice-mismatch and possible contaminations at the interface^51^. For MoS_2_, Taube *et al*. measured the total G across MoS_2_/Si including a thin SiO_2_ layer to be 1.94 MW/m^2^K^41^, and Zhang *et al*. measured G to be 0.44 MW/m^2^K for MoS_2_/Au^40^. These results were obtained by solving the heat diffusion equation based on the temperatures obtained from Raman spectroscopy, and the thermal conductivity was obtained simultaneously. However, this method requires the precise determination of absorbed laser power, to which interface thermal conductance and the thermal conductivity are sensitive.

In this work, we directly measure and compare G between MoS_2_/*h*-BN and graphene/*h*-BN, borrowing the technique from the work of Yue *et al*.^50^ and Chen *et al*.^51^, while paying special attention to the interface quality. Specifically, we increase the temperature of the top MoS_2_ (graphene) through Joule heating, and at the same time, monitor the temperatures of MoS_2_ (graphene) and *h*-BN via Raman spectroscopy. The thermal conductance across the MoS_2_ and *h*-BN interface is (17.0 ± 0.4) MW/m^2^K, which is ~3 times smaller than G for graphene/*h*-BN interface measured as (52.2 ± 2.1) MW/m^2^K. With the expected lower in-plane thermal conductivity of MoS_2_ compared to graphene, this could indicate that electronic and optoelectronic devices using MoS_2_/*h*-BN have a larger propensity for failure due to overheating and therefore this value needs to be considered carefully in the future design of such devices.

To understand further the physics behind this difference in G, we conduct non-equilibrium Green’s function (NEGF) calculations of these two interfaces, which show a trend consistent with experiments. Our simulation consists of a semi-infinite superstrate of graphene or MoS_2_ sheets and a semi-infinite substrate of *h*-BN sheets. The graphene layers are stacked in the A-B configuration while the *h*-BN and MoS_2_ are stacked in the A-A’ configuration. In each structure, the interfacial area is 2.49 × 2.2 nm^2^. The interatomic potentials are obtained from literature^53^,^54^,^55^. After optimizing the structures in GULP^56^, we compute their force constant matrices and input them into our NEGF code to calculate the transmission spectrum across the interface. The interface thermal conductance G is obtained using the formula


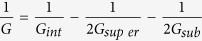


where *G*_*int*_, *G*_*super*_ and *G*_*sub*_ are respectively the conductance of the MoS_2_/*h*-BN (or graphene/*h*-BN), MoS_2_/MoS_2_ (or graphene/graphene) and *h*-BN/*h*-BN interfaces.

## Results

**Figure 1:**
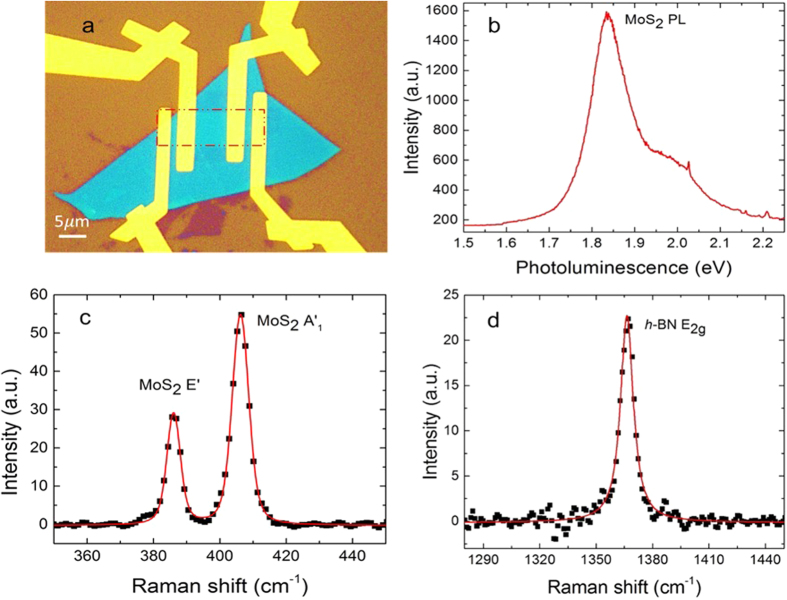
(**a**) Optical image of the MoS_2_/*h*-BN heterostructure. Dashed box indicates MoS_2_. (**b**) Photoluminescence and (**c**) Raman bands of MoS_2_. The PL location at 1.84 eV and the distance between the *E*′ and the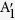 band (~18 cm^−1^) indicates that it is indeed monolayer MoS_2_. (**d**) Raman *E*_*2g*_ band indicating the high quality of *h*-BN.

The fabricated MoS_2_/*h*-BN FET device is shown in Fig. 1(a). The Raman spectra of MoS_2_ and *h*-BN are shown in Fig. 1(c) and (d). The photoluminescence (PL) of MoS_2_ is shown in Fig. 1(b). The distance between the MoS_2_ *E*′ peak and the 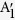 peak (~18 cm^−1^)^57^, and the location of the PL peak at 1.84 eV indicate that our sample is indeed monolayer MoS_2_^15^. The four electrodes are patterned in order to probe the electrical contact resistance (R_c_) between MoS_2_ and the two inner electrodes, which are used for Joule heating.

To determine G, it is necessary to know the temperatures of the MoS_2_ and *h*-BN. Raman spectroscopy has been demonstrated to be an effective method to probe the temperatures of 2D materials^31^,^33^,^34^,^37^,^40^,^41^,^58^, based on the fact that the Raman spectra red-shifts as temperature rises due to lattice softening and anharmonic scattering of phonons. To measure temperatures with Raman spectroscopy, the experiment is divided into two parts. The first part is the calibration of the Raman shifts for MoS_2_ and *h*-BN as a function of temperature. At this stage, the temperatures of the materials are externally controlled via a heater stage, and their corresponding Raman spectra are collected at each temperature point. This calibration ensures that the temperature of each material can be determined by referring to their Raman spectra in the subsequent Joule heating measurement. Next, a series of current bias are applied to the electrically conducting MoS_2_ to provide Joule heat power given by *P*_*o*_ = *I*^*2*^*R* (where *I* is the drain source current and *R* is the electrical resistance of MoS_2_ including contact resistance *R*_*c*_). The laser spot is located in the middle of the sample. Note here that the relaxation time for electron-phonon interaction is on a short time scale of picoseconds with a relaxation length of the order of nanometers^59^, while our measurements are in steady state for a sample size of several microns, and thus there should be no discrepancy between Joule heating and global cryostat heating. The generated heat is dissipated in the cross-plane direction and heats up the underlying *h*-BN, while the sample stage is maintained at room temperature. All measurements are performed in a high vacuum environment in a closed-cycle cryostat (<10^−5^ torr). The Raman spectra of MoS_2_ and *h*-BN are monitored at each current bias to determine their temperatures based on the calibration results in the first part of measurement.

**Figure 2:**
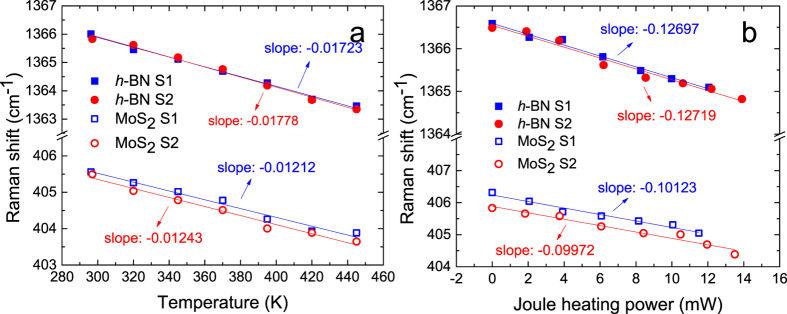
Raman measured results for MoS_2_/*h*-BN interface. (**a**) Temperature calibration of Raman shift. (**b**) Raman shift of MoS_2_ 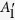 peak, and *h*-BN as a function of Joule heating power of MoS_2_: as the power increases, the temperature also increases, indicated as Raman red-shifts.

The temperature calibration of the Raman spectra of MoS_2_ and *h*-BN are shown in Fig. 2(a). For MoS_2_, the in-plane *E*′ mode is sensitive to strain, while the out-of-plane 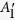 mode is not^60^,^61^. Thus, the 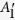 peak is chosen as the temperature indicator for MoS_2_ to avoid interference from strain-induced Raman shifts. The relationship between the Raman shift and the temperature can be expressed as 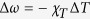, where Δ*ω* is the Raman shift due to temperature change, *χ*_*T*_ is the first-order temperature coefficient, and Δ*T* is the temperature change. The *χ*_*T*_ for the MoS_2_ 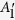 peak and *h*-BN are measured to be 0.01212 cm^−1^ · K^−1^ and 0.01723 cm^−1^·K^−1^ for the first sample, and 0.01243 cm^−1^ · K^−1^ and 0.01778 cm^−1^ · K^−1^ for the second sample, respectively.

During the MoS_2_ Joule heating process, the temperatures of the MoS_2_ and the underlying *h*-BN increase, resulting in red shifts of thr Raman peaks as indicated in Fig. 2(b). The Joule heating power coefficient *χ*_*P*_, which is defined as 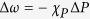, is measured to be 0.10123 cm^−1^ · mW^−1^ and 0.12697 cm^−1^ · mW^−1^ for the MoS_2_ 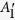 peak and *h*-BN for the first sample, and 0.09972 cm^−1^ · mW^−1^ and 0.12719 cm^−1^ · mW^−1^ for the MoS_2_ 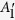. peak and *h*-BN for the second sample.

The interface thermal conductance between MoS_2_ and *h*-BN is calculated from


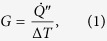


where Δ*T* is the temperature difference beten MoS_2_ and *h*-BN.


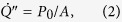


is the heat flux across the interface. Here, *A* is the interface area (including the transfer lengths from contacts) between MoS_2_ and *h*-BN, which is 50.50 μm^2^ and 73.05 μm^2^ for the two samples we measured. *P*_*O*_ = *P* + *P*_*c*_ is the total Joule heating power, including both the power *P* from the MoS_2_ channel and *P*_*c*_ from the contact resistance *R*_*c*_ between MoS_2_ and the two inner electrodes used for Joule heating. *P*_*c*_ is determined through a 2-probe/4-probe resistance measurement, and 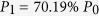 and 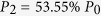 for the two samples is found (Supplementary Information). Δ*T* can be obtained based on the Raman measurements described above, i.e.,


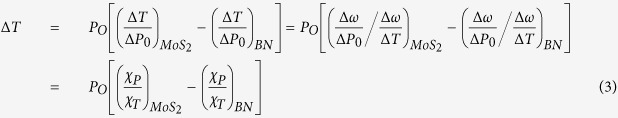


Combining (1) and (2), the interface thermal conductance G is calculated to be 20.1 MW · m^−2^K^−1^ and 15.8 MW · m^−2^K^−1^ for the two samples.

To improve the accuracy of our measured G, we also consider the non-uniform heat flux within the basal plane of MoS_2_ and the influence from the contact resistance. A three-dimensional finite element method (3D FEM) using COMSOL is used to estimate the *local* (center of the sample) heat flux. This is realized by treating G as an input parameter and iteratively making it consistent with the local temperature difference observed from experiments (Supplementary information). The G for these two samples after local heat flux correction is 17.3 MW · m^−2^K^−1^ and 16.7 MW · m^−2^K^−1^. By averaging the values of G from the two samples after 3D FEM correction, we obtain the effective interface thermal conductance between MoS_2_ and *h*-BN of (17.0 ± 0.4) MW · m^−2^K^−1^. The G obtained for MoS_2_/*h*-BN interface here is larger than that of the MoS_2_/Au interface which has a value of 0.44 MW · m^−2^K^−1^ 40. This is possibly because of the ultra-smooth *h*-BN surface and/or better thermal coupling between MoS_2_ and *h*-BN intrinsically.

**Figure 3:**
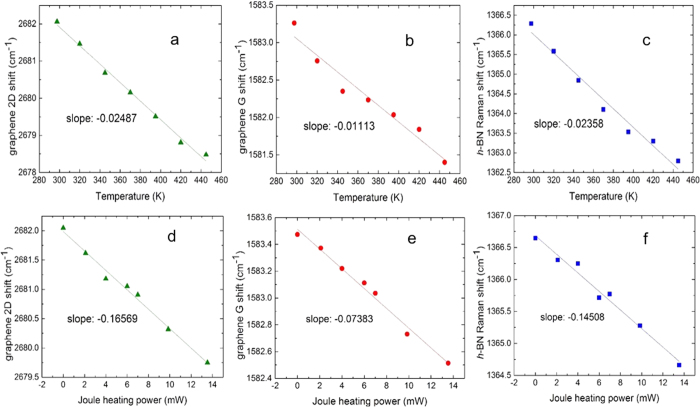
Raman measured results for graphene/*h*-BN interface. Temperature calibration of Raman shift of graphene (**a**) 2D band and (**b**) G band, and (**c**) *h*-BN. Raman shift of graphene (**d**) 2D band and (**e**) G band, and (**f**) *h*-BN as the Joule heating power of graphene increases, indicating temperature rises.

To obtain a better understanding of thermal conductance across such van der Waals interfaces, we also measure G for the graphene/*h*-BN interface for comparison. Similarly, the measurement is composed of two parts, that is, the Raman shift calibration against temperature and the Joule heating measurement. The measurement results for the two parts are shown in Fig. 3. For the graphene/*h*-BN interface, with an interface area of 43.55 μm^2^ and 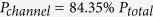, the thermal conductance is extracted to be 45.1 MW · m^−2^K^−1^ and 47.8 MW · m^−2^K^−1^ based on the graphene Raman G band and 2D band respectively. Similar to the MoS_2_/*h*-BN case, the local heat flux correction from 3D FEM COMSOL simulation gives 53.7 MW · m^−2^K^−1^ and 50.7 MW · m^−2^K^−1^ based on the graphene Raman G band and 2D band respectively, resulting in an average effective value of (52.2 ± 2.1) MW · m^−2^K^−1^. When observing the heterostructures with AFM, we find bubbles of size ~100 nm randomly dispersed within the interfaces of both MoS_2_/*h*-BN and graphene/*h*-BN (Supplementary information). These bubbles are almost unavoidable during the transfer process, and difficult to remove completely even after annealing. It has been shown that these bubbles could be formed by chemical adsorbates on crystals used for preparing the heterostructures in ambient conditions, and the trapped materials could be amorphous hydrocarbons^62^. Depending on the thermal conductance of the bubbles: if bubbles have a smaller/larger G than the intrinsic G between MoS_2_/*h*-BN, our measurement results represents the lower/upper bounds of G. Bubble-free 2D heterostructures could be accessible with fabrication processes performed in a vacuum environment, which deserves further studies.

Compared with the G of 7.4 MW · m^−2^K^−1^ measured in Chen *et al*.^51^, our observed higher G for the graphene/*h*-BN interface is due to the higher quality of the interfaces. Our G for the graphene/*h*-BN interface is also much higher than that of the graphene/SiC interface (0.0187 MW · m^−2^K^−1^)^50^. This is possibly due to less strain in the graphene superstrate when it is van der Waals coupled with *h*-BN than when it is covalent bonded to SiC, as tensile strain in graphene during the heating process tends to increase the red-shift of Raman peaks, resulting in the overestimation of the temperature of graphene and thereby underestimates G.

**Figure 4:**
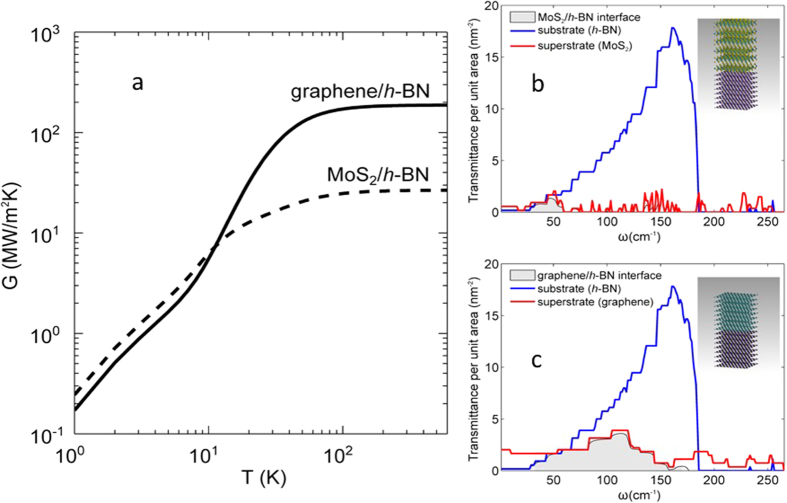
(**a**) Temperature dependence of the thermal conductance of the MoS_2_/*h*-BN and the graphene/*h*-BN interface. (**b**) and (**c**) show the schematic of the atomistic structure of the interface and its transmittance spectrum along with the spectra of the superstrate and the substrate for the MoS_2_/*h*-BN and the graphene/*h*-BN interfaces, respectively. The transmittance per unit area indicates the number of phonons transmitted across the interface per unit area.

Compared with the MoS_2_/*h*-BN interface, graphene/*h*-BN has a better interface thermal conductance. To better understand the physics behind this difference, we employ the NEGF technique to calculate the thermal conductance and transmittance spectrum. Figure 4 shows the temperature dependence of the thermal conductance and transmittance spectrum for the MoS_2_/*h*-BN and the graphene/*h*-BN interfaces. Our measurement results of G (17.0 ± 0.4) is close to that from NEGF calculation of 26.4 MW · m^−2^K^−1^, which serves as the theoretical upper bound of this interface, indicating the high quality of our interface. The measured G for the graphene/*h*-BN (52.2 ± 2.1) MW · m^−2^K^−1^ is larger than that for MoS_2_/*h*-BN, the same trend as predicted by NEGF calculations. From the phonon transmittance spectrum across the interfaces [Fig. 4(b) and (c)], it can be observed that the lower G across the MoS_2_/*h*-BN interface is due to the lower phonon transmittance of MoS_2_, which has a much smaller overlap with the *h*-BN transmittance spectrum and thus, a smaller effective transmittance spectrum for the interface, as compared to the graphene/*h*-BN case. Thus, the much lower thermal conductance for the MoS_2_/*h*-BN interface is due to the phonon transmission bottleneck caused by the limited number of transmission channels in MoS_2_, compared to the greater number of transmission channels in graphene.

Although G for graphene/*h*-BN interface is larger than that of the MoS_2_/*h*-BN interface, it is still much smaller than the NEGF-calculated values. This may be due to tensile strain originating from thermal expansion in graphene, which tends to increase the Raman peak red-shift^63^,^64^,^65^ during the Joule heating process, thus overestimating the temperature of graphene, and finally underestimating G based on eq. (1). It is worth noting that this effect of strain does not affect significantly the measurement of the MoS_2_/*h*-BN interface, as we intentionally chose the strain-insensitive 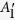 mode of MoS_2_ as the temperature indicator. Therefore, we observed G for the MoS_2_/*h*-BN interface to be closer to theoretical predictions. Another explanation for the discrepancy is the dependence of G on the number of layers which has been shown in molecular dynamics simulations^66^ to be lower for a single-layer crystal than for a multilayer crystal. Hence, it is expected that the interface thermal conductance for multilayer structures should be significantly higher than for single-layer 2D crystals. Our measured G values are for temperatures higher than 300 K (the stage is kept at 300 K while the top MoS_2_ or graphene is Joule heated with the temperature higher than 300 K), and G should be temperature-independent above this temperature, based on NEGF calculations (Fig. 4). For the MoS_2_/*h*-BN interface in particular, heat is dissipated primarily via the low-frequency (sub-70 cm^−1^) modes whose contributions to the conductance are temperature-independent when *T* ≫ 100 K.

## Discussion

In summary, we have measured the thermal conductance across the interface of 2D MoS_2_/*h*-BN and graphene/*h*-BN heterostructures. This is realized by Joule heating the MoS_2_ (graphene) superstrate, and monitoring the temperatures of both the superstrate MoS_2_ (graphene) and *h*-BN substrate through Raman spectroscopy. The measured thermal conductance of MoS_2_/*h*-BN interface is (17.0 ± 0.4) MW · m^−2^K^−1^, which is smaller than the value of graphene/*h*-BN interface (52.2 ± 2.1) MW · m^−2^K^−1^, consistent with the trend predicted from our NEGF calculations. We attribute the lower thermal conductance across the MoS_2_/*h*-BN interface to the limited cross-plane phonon transmission in MoS_2_, when compared to graphene. Our measurements and calculations provide basis for the study of in-plane thermal conductivity of MoS_2_ supported by *h*-BN, the typical structure for building high-mobility TMDC FET devices. The low interface thermal conductance deserves careful consideration for the design of electronic and optoelectronic devices based on such heterostructures.

## Methods

The MoS_2_ flake was exfoliated onto a 285 nm SiO_2_/Si substrate, and was confirmed to be monolayer from its Raman distance between the *E*′ and 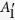 peaks (~18 cm^−1^) photoluminescence peak (~1.84 eV) (Fig. 1). The *h*-BN flake was exfoliated onto another 285 nm SiO_2_/Si substrate. The MoS_2_ was then transferred onto the *h*-BN with polymethyl methacrylate (PMMA) as an assisted layer followed by acetone etching. The electrodes were patterned by standard electron-beam lithography (EBL) process, followed with thermal evaporation of Ti/Au with thickness of 5 nm and 75 nm, respectively. The sample was then annealed *in*-*situ* in vacuum at 400 K for 6 hours to release strains produced during the transfer process and ensure that the two materials are fully adhered^67^. The graphene/*h*-BN heterostructure was prepared following the same procedure.

After measurements, the interface quality was characterized with AFM. For MoS_2_/*h*-BN, bubbles of the size of ~100 nm were found at the interface. We found that these bubbles are almost unavoidable during the transfer process and difficult to remove once formed. Generally, high temperature annealing (350 °C) tends to make small bubbles from larger ones for graphene/*h*-BN^62^. Therefore, we annealed the graphene/*h*-BN samples in Ar atmosphere at 350 °C for 3.5 hours, and specifically chose areas with fewer bubbles for device fabrication. For those regions without bubbles, we found the roughness of the surface, which is defined as the root mean square average of height deviations taken from the mean image data plane, is ~1.5 nm, with the main contribution from polymeric residues during the transfer process. These polymeric residues are on the top surface of MoS_2_, thus won’t affect the interface. We find no obvious difference in roughness for the surface of *h*-BN and the region of MoS_2_ without bubbles, indicating the good conformity of the interface.

## Additional Information

**How to cite this article:** Liu, Y. *et al*. Thermal Conductance of the 2D MoS_2_/*h*-BN and graphene/*h*-BN Interfaces. *Sci. Rep.* **7**, 43886; doi: 10.1038/srep43886 (2017).

**Publisher's note:** Springer Nature remains neutral with regard to jurisdictional claims in published maps and institutional affiliations.

## Supplementary Material

Supplementary Information
