# Supplementary material for: Thermal Conductance of the 2D MoS2/h-BN and graphene/h-BN Interfaces
Source: Sci Rep. 2017 Mar 6;7:43886. doi: 10.1038/srep43886 (PMC5338337; doi:10.1038/srep43886)
Supplement: Supplementary Information [file srep43886-s1.pdf]

Supplementary Information for Thermal Conductance of the 2D MoS_2/*h*-BN and graphene/*h*-BN Interfaces

Yi Liu[1], Zhun-Yong Ong[2], Jing Wu[3], Yunshan Zhao[1], Kenji Watanabe[4], Takashi Taniguchi[4], Dongzhi Chi[3], Gang Zhang[2], John TL Thong[1]*, Cheng-Wei Qiu[1,5]*, Kedar Hippalgaonkar[3]*

[1]Department of Electrical and Computer Engineering, National University of Singapore, Engineering Drive 3, Singapore 117583
[2]Institute of High Performance Computing, #16-16, 1 Fusionopolis Way, Agency for Science, Technology and Research, Singapore, 138632
[3]Institute of Materials Research and Engineering, #08-03, 2 Fusionopolis Way, Agency for Science, Technology and Research, Singapore, 138634
[4]National Institute for Materials Science, Tsukuba, Ibaraki, Japan, 305-0044
[5]Optical Science and Engineering Center, Department of Electrical and Computer Engineering, National University of Singapore, Singapore 117583
*Kedar Hippalgaonkar: kedarh@imre.a-star.edu.sg
*John TL Thong: john_thong@nus.edu.sg
*Cheng-Wei Qiu: chengwei.qiu@nus.edu.sg

1. COMSOL simulation

Considering the non-uniform heat flux across the MoS_2 (graphene)/ *h*-BN and the influence from the contacts between MoS_2 (graphene) and the electrodes, we performed a three-dimensional COMSOL simulation to correct the thermal interface conductance (G) calculated in equation (1) and (2) in the main text, which is based on the assumption of uniform heat flux. The idea is to iteratively obtain a final value of G that makes the local temperature differences across heterostructure equal to that is measured in our experiments (**Figure. S1**). The simulation is built based on the real geometries and contact resistances of the samples. The input parameters are listed in **Table. S1**. Considering the contact resistance, we assigned the total Joule heating power P_0 to the channel and the contacts based on the resistance ratio R_{4p}/R_{2p}. That is, $P_0*(R_{4p}/R_{2p})$ is assigned to be generated by the MoS_2 or graphene channel, and $P_0*(1-R_{4p}/R_{2p})$ is assigned to be generated by the contacts between metal and MoS_2 or graphene. The results of this iterative process are listed in **Table S2**. The temperature profile of the MoS_2/*h*-BN sample 1 is demonstrated in **Figure S2** as an example.

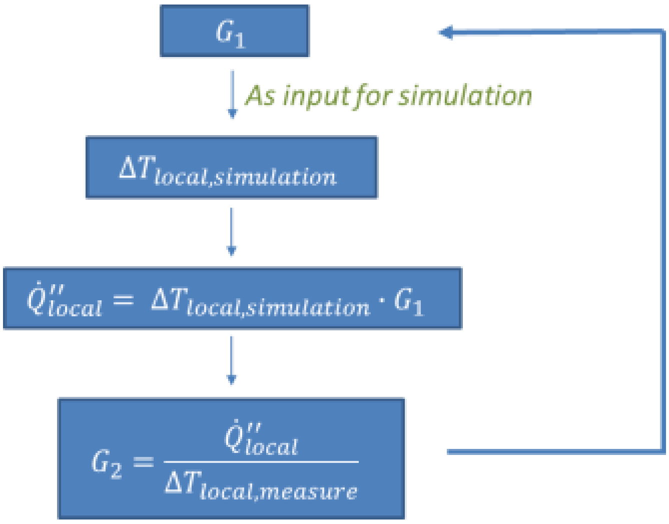

Figure. S1. Work flow for iterative calculation of G based on COMSOL simulation.

**Table. S1 Input parameters in COMSOL simulation**								
k_{MoS2} (W/mK)	$k_{graphene}$ (W/mK)	$k_{h\text{-}BN}$ (W/mK)		$G_{MoS2/Au}$ (MW/m^{2}K)	$G_{h\text{-}BN/SiO2}$ (MW/m^{2}K)	Cp_{MoS2} (J/kgK)	$Cp_{graphene}$ (J/kgK)	$Cp_{h\text{-}BN}$ (J/kgK)
62.2[1]	370[2]	a-axis	c-axis	0.44[3]	45.45[4]	398.13[5]	538.33[6]	806.35[7]
		360[8]	30[9]					

* k denotes thermal conductivity; G denotes Interface thermal conductance; C_p denotes heat capacity at constant pressure.

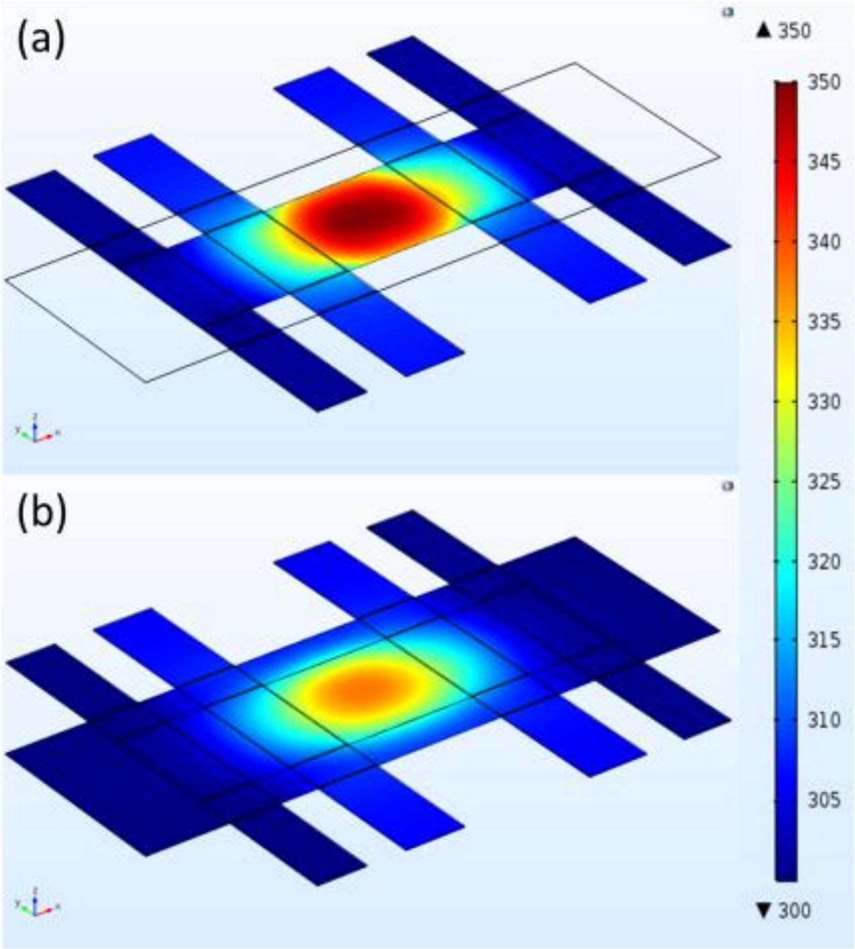

Figure. S2 Temperature profiles of (a) MoS_2 and (b) *h*-BN from COMSOL simulation.

Table. S2 Iterative results from simulation

MoS_2/*h*-BN sample 1

Cycle number	1	2	3	4
G1 (MW·m^{-2}K^{-1})	20.14	17.174411	17.2565	17.25341
ΔTlocal,simulation (K)	10.06076	11.85439	11.79589	11.79808
$\dot{Q}''_{local}$ (MW·m^{-2})	202.6237064	203.5922	203.5558	203.5571
G2 (MW·m^{-2}K^{-1})	17.174411	17.2565	17.25341	17.25353

1) MoS_2/*h*-BN sample 2

Cycle number	**1**	**2**	**3**
G1 (MW·m^{-2}K^{-1})	15.75	16.75159	16.72852
$\Delta T_{local,simulation}$ (K)	12.93942	12.149	12.16612
$\dot{Q}''_{local}$ (MW·m^{-2})	203.795865	203.5151	203.5211
G2 (MW·m^{-2}K^{-1})	16.75159	16.72852	16.72901

2) Graphene/*h*-BN results based on graphene 2D band

Cycle number	**1**	**2**	**3**
G1 (MW·m^{-2}K^{-1})	45.06	50.65039	50.67912
$\Delta T_{local,simulation}$ (K)	7.90454	7.03609	7.03212
$\dot{Q}''_{local}$ (MW·m^{-2})	356.1785724	356.3807	356.3817
G2 (MW·m^{-2}K^{-1})	50.65039	50.67912	50.67927

3) Graphene/*h*-BN results based on graphene G band

Cycle number	1	2	3
G1 ($MW\cdot m^{-2}K^{-1}$)	47.76	53.69173	53.73729
$\Delta T_{local,simulation}$ (K)	7.45838	6.64	6.63477
$\dot{Q}''_{local}$ ($MW\cdot m^{-2}$)	356.210796	356.5131	356.5346
G2 ($MW\cdot m^{-2}K^{-1}$)	53.69173	53.73729	53.74053

2. Bubbles and relationship with G

We find that bubbles are formed and almost unavoidable during the transfer process. To examine the morphology of the bubbles, AFM is carried out on both MoS_2/*h*-BN and graphene/*h*-BN samples with the height images shown in **Figure S3**. Hence, we report an 'effective' value of thermal conductance, which includes any thermal conductance through the bubbles. The measured G depends on the interface quality as well as intrinsic thermal properties. To consider the relationship between G and bubble density for a fair comparison of G between different samples, we link the bubble density with G as follows.

a. Bubble density

The bubble densities can be characterized with AFM. Typical morphology of the MoS_2/*h*-BN and graphene/*h*-BN is shown in Fig. S3. Regions are defined as bubbles when their heights are larger than the roughness (root mean square Rq ~1.5 nm) of *h*-BN. The reason for choosing this cut-off height is that the roughness of sample surfaces comes from two parts: (1) bubbles between the 2D superstrate (either MoS_2 or graphene) and the substrate *h*-BN and (2) PMMA residues on the top of the 2D superstrate from the transfer process (note that the bottom surface of the MoS_2/graphene as well as the top surface of the *h*-BN of the heterostructures do not have any PMMA residue). Based on bearing analysis in the NanoScope Analysis software, which reveals how much of a surface lies above or below a given height, the bubbles take up to 28.7% and 13.9% for MoS_2/*h*-BN and graphene/*h*-BN interfaces, respectively. It is to be noted that this estimate is conservative, as the AFM roughness includes any additional PMMA residue on top of the superstrate, which does not affect the thermal interface conductance between the superstrate and substrate.

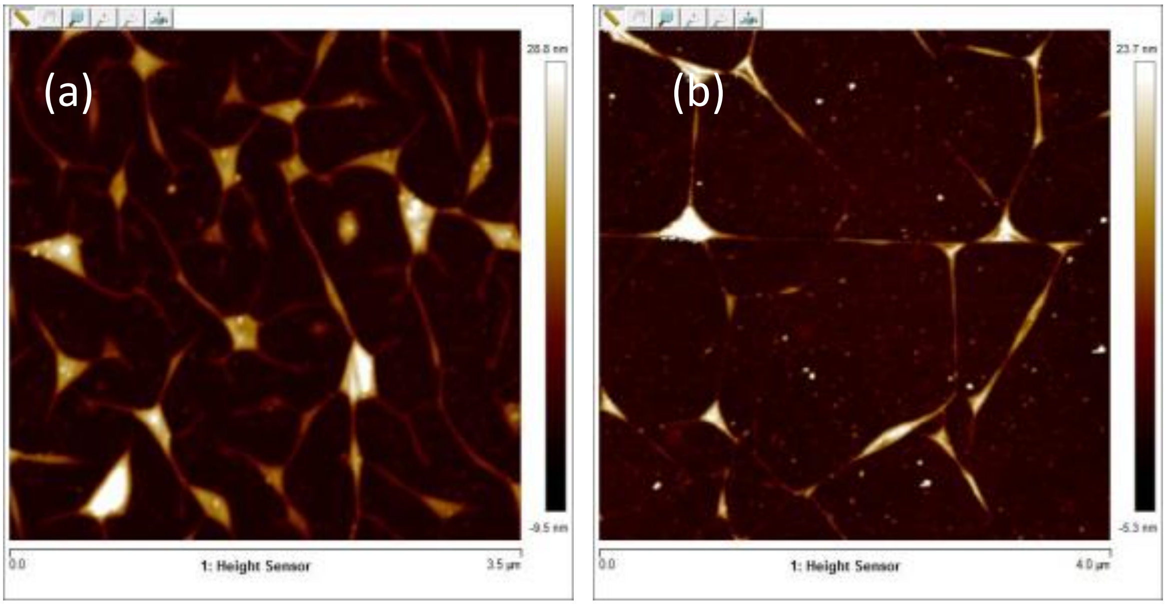

Figure. S3. AFM height images of MoS_2/*h*-BN (a) and graphene/*h*-BN (b) samples, with the bubble density of 28.7% for MoS_2/*h*-BN, and 13.9% for graphene/*h*-BN, respectively.

b. Relationship between G and bubble density

With the bubble density defined as A_{bubble}/A_{total}, where A denotes 'area', the intrinsic thermal conductance $G_{intrinsic}$ can be estimated based on the measured G_{total} by the relationship of

$$G_{total}A_{total} = G_{intrinsic}A_{intrinsic} + G_{bubble}A_{bubble}\ , \qquad \text{(S1)}$$

where $A_{total} = A_{intrinsic} + A_{bubble}$ is the total contacting area, including the good contact area $A_{intrinsic}$, and the bubble area A_{bubble}.

We note that if the bubble gives a lower G than an intrinsic G, the reason of lower G in MoS_2/*h*-BN than that in graphene/*h*-BN could be the bubble density. To clarify this, based on the bubble density of A_{bubble}/A_{total} from the AFM analysis above, even if we assume bubbles have a lower G than the intrinsic interfaces, for instance, $G_{bubble} = 0.1*G_{intrinsic}$, $G_{intrinsic}$ can be estimated as 22.9 MW/m^2K for MoS_2/*h*-BN, which is still much smaller than 59.7 MW/m^2K for graphene/*h*-BN. Actually, as the bubbles take up less than ¼ of the total contact area, the thermal conductance through the bubbles doesn't contribute substantially to the measured G_{total}. In the worst case where $G_{bubble} = 0$, we still have $G_{MoS2/h\text{-}BN} = 23.8$ MW/m^2K much smaller than $G_{graphene/h\text{-}BN} = 60.6$ MW/m^2K, confirming our measurement that *intrinsically* the MoS_2/*h*-BN interface has a lower G than that of graphene/*h*-BN.

3. Electrical contact resistance

To obtain the electrical contact resistances, 2-probe/4-probe measurements were conducted (**Figure S4**). The measurements gave R_{4p}/R_{2p} = 70.19%, R_{4p}/R_{2p} = 53.55%, and R_{4p}/R_{2p} = 70.19% for the three samples (2 MoS_2/*h*-BN samples, 1 graphene/*h*-BN sample).

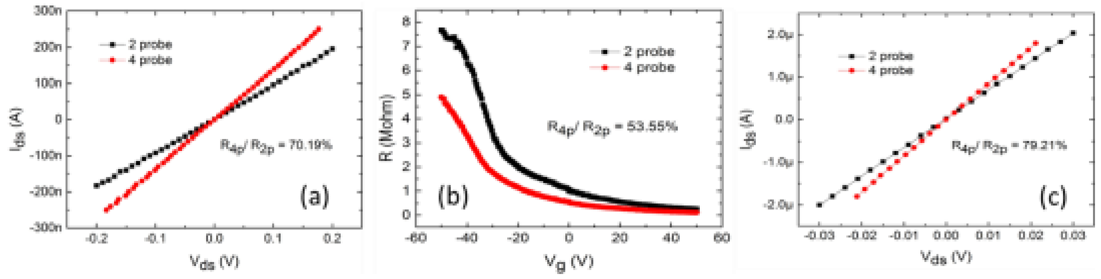

Figure S4. 2-probe/4-probe electrical measurement of MoS_2/*h*-BN (a) sample 1 and (b) sample 2, and (c) graphene/*h*-BN sample.

4. NEGF simulation

We simulate the heat flux between a semi-infinite superstrate of graphene (or MoS_2) sheets and a semi-infinite substrate of *h*-BN sheets. The graphene layers are stacked in the A-B configuration while the *h*-BN and MoS_2 are stacked in the A-A' configuration. In each structure, the interfacial area is 2.49×2.16 nm^2. In the directions orthogonal to the direction of the heat flux, we impose periodic boundary conditions. The interatomic potentials in the structures are taken from Refs.[10–12] After optimizing the structures in GULP,[13] we compute their force constant matrices which we use to calculate the transmission spectrum and thermal conductance of the interface. The thermal conductance is given by the formula[14]

$$G(T) = \frac{1}{2\pi S}\int_0^\infty d\omega\, \hbar\omega \frac{dN}{dT}\Xi(\omega)$$

where S is the interfacial area, N is the Bose-Einstein distribution at temperature T, and $\Xi(\omega)$ is the transmittance at frequency ω. The transmittance is given by the formula $\Xi(\omega) = Tr[G^r\Gamma_L G^a\Gamma_R]$, where G^r and G^a are respectively the retarded and advanced Green's function of the central region (the interface) connected to the two leads. The Green's functions are defined by $G^r = (G^a)^\dagger = [\omega^2 - K_C - \Gamma_L - \Gamma_R]^{-1}$, where K_C is the mass-normalized force constant matrix for the central region. Γ_L (Γ_R) is the broadening function corresponding to the left (right) lead and is given by $\Gamma_{L,R} = i(\Sigma^r_{L,R} - \Sigma^a_{L,R})$ where $\Sigma^r_{L,R}$ ($\Sigma^a_{L,R}$) is the retarded (advanced) self-energy associated with the left or right lead.

To calculate the thermal conductance of the interface, we calculate the conductances G_{super} for the graphene/graphene (or MoS_2/MoS_2) interface, G_{sub} for the *h*-BN/*h*-BN interface and G_{int} for the graphene/*h*-BN (or MoS_2/*h*-BN) interface. The interface thermal conductance (G) is calculated using the formula[15,16]

$$\frac{1}{G} = \frac{1}{G_{int}} - \frac{1}{2G_{super}} - \frac{1}{2G_{sub}}$$

References

1. Taube, A., Judek, J., Łapińska, A. & Zdrojek, M. Temperature-dependent thermal properties of supported MoS2 monolayers. *ACS Appl. Mater. Interfaces* **7,** 5061–5065 (2015).
2. Cai, W. *et al.* Thermal transport in suspended and supported monolayer graphene grown by chemical vapor deposition. *Nano Lett.* **10,** 1645–1651 (2010).
3. Zhang, X. *et al.* Measurement of Lateral and Interfacial Thermal Conductivity of Single- and Bilayer MoS2 and MoSe2 Using Refined Optothermal Raman Technique. *ACS Appl. Mater. Interfaces* **7,** 25923–25929 (2015).
4. Ni, Y., Jiang, J., Meletis, E. & Dumitricə, T. Thermal transport across few-layer boron nitride encased by silica. *Appl. Phys. Lett.* **107,** 31603 (2015).
5. Volovik, L. S. *et al.* Enthalpy and heat capacity of molybdenum disulfide. *Sov. Powder Metall. Met. Ceram.* **17,** 697–702 (1978).
6. Ma, F. *et al.* Strain effect on lattice vibration, heat capacity, and thermal conductivity of graphene. *Appl. Phys. Lett.* **101,** 111904 (2012).
7. Dworkin, a. S., Sasmor, D. J. & Van Artsdalen, E. R. The Thermodynamics of Boron Nitride; Low-Temperature Heat Capacity and Entropy; Heats of Combustion and Formation. *J. Chem. Phys.* **22,** 837–842 (1954).
8. Jo, I. *et al.* Thermal conductivity and phonon transport in suspended few-layer hexagonal boron nitride. *Nano Lett.* **13,** 550–554 (2013).
9. Duclaux, L., Nysten, B., Issi, J.-P. & Moore, A. W. Structure and Low Temperature Thermal Conductivity of Pyrolytic Boron Nitride. *Phys. Rev. B* **46,** 3362–3368 (1992).
10. Ding, Z., Jiang, J.-W., Pei, Q.-X. & Zhang, Y.-W. In-plane and cross-plane thermal conductivities of molybdenum disulfide. *Nanotechnology* **26,** 65703 (2015).
11. Kınacı, A., Haskins, J. B., Sevik, C. & ÇağIn, T. Thermal conductivity of BN-C nanostructures. *Phys. Rev. B* **86,** 115410 (2012).
12. Ye, Z., Otero-de-la-Roza, A., Johnson, E. R. & Martini, A. Oscillatory motion in layered materials: graphene, boron nitride, and molybdenum disulfide. *Nanotechnology* **26,** 165701 (2015).
13. Gale, J. D. GULP: A computer program for the symmetry-adapted simulation of solids. *J. Chem. Soc., Faraday Trans.* **93,** 629–637 (1997).
14. Zhang, W., Fisher, T. S. & Mingo, N. The Atomistic Green's Function Method: An Efficient Simulation Approach for Nanoscale Phonon Transport. *Numer. Heat Transf. Part B Fundam.* **51,** 333–349 (2007).
15. Serov, A. Y., Ong, Z.-Y. & Pop, E. Effect of grain boundaries on thermal transport in graphene. *Appl. Phys. Lett.* **102,** 33104 (2013).
16. Tian, Z., Esfarjani, K. & Chen, G. Enhancing phonon transmission across a Si/Ge interface by atomic roughness: First-principles study with the Green's function method. *Phys. Rev. B - Condens. Matter Mater. Phys.* **86,** 235304 (2012).
